# Case Report: Dose-escalated volumetric modulated arc therapy achieving bowel and renal protection for large retroperitoneal inflammatory myofibroblastic tumor invading ureter and inferior vena cava

**DOI:** 10.3389/fonc.2026.1818998

**Published:** 2026-08-11

**Authors:** Jiarui Zhang, Tianhao Liu, Cuihua Zhang, Siming Shi, Peng Zhen, Fang Fang

**Affiliations:** 1 Department of Radiation Oncology, Chifeng Tumor Hospital, Chifeng, Inner Mongolia Autonomous Region, China; 2 Department of Pathology, Chifeng Tumor Hospital, Chifeng, Inner Mongolia Autonomous Region, China

**Keywords:** granuloma, plasma cell, organ sparing treatments, radiotherapy dosage, radiotherapy, intensity-modulated, retroperitoneal neoplasms

## Abstract

Inflammatory myofibroblastic tumor (IMT) is a rare mesenchymal neoplasm for which definitive radiotherapy in the retroperitoneum remains largely undefined, particularly when dose escalation must be balanced against the preservation of adjacent critical organs. A 59-year-old woman presented with a 10-day history of fever and abdominopelvic pain. Physical examination revealed deep tenderness in the right lower quadrant, and laboratory testing demonstrated leukocytosis. Imaging identified a 9.2 cm retroperitoneal mass invading the right ureter and inferior vena cava. Biopsy confirmed IMT, and capture-based next-generation sequencing revealed no class I actionable alterations. Given the unfavorable surgical anatomy and limited response to corticosteroids, curative-intent resection was precluded. The patient was treated with definitive dose-escalated volumetric modulated arc therapy (VMAT) employing a simultaneous integrated boost strategy (66 Gy/33 fractions to the boost volume and 50 Gy to the conventional planning target volume), with spatial sparing of adjacent organs at risk. Complete radiographic response was achieved at one month, without grade ≥2 treatment-related adverse events. At 19-month follow-up, the patient remained disease-free with stable renal function. This case demonstrates that dose-escalated VMAT with spatial organ avoidance can achieve durable complete remission in unresectable retroperitoneal IMT while preserving organ function, supporting its consideration as a curative-intent strategy in this challenging clinical setting.

## 1 Introduction

Inflammatory myofibroblastic tumor (IMT) is a rare mesenchymal neoplasm composed of myofibroblastic spindle cells accompanied by a lymphoplasmacytic infiltrate (1). IMT can occur throughout the body, with the lung being the most common site; however, it has also been documented in the head and neck, thoracic, abdominopelvic cavities, extremities (2), and the retroperitoneum represents one of the potential locations (3). The clinical presentation of IMT is nonspecific, with symptoms varying according to the anatomical site involved. Patients may experience systemic symptoms such as fever and weight loss (4). IMT generally carries a favorable prognosis, with a metastatic risk of less than 5% following curative-intent treatment (5); however, the recurrence rate is approximately 25% (4). Studies have indicated that a tumor size of ≥8 cm is suggestive of aggressive tumor behavior and predicts a higher risk of recurrence (6). Surgical resection is often the primary treatment modality (7). However, for patients who are not candidates for curative surgery or cannot tolerate a surgical procedure, radiotherapy constitutes an effective treatment option. Currently, no standardized consensus exists regarding the optimal radiotherapy regimen and dosage. Theoretically, larger tumor volumes necessitate higher prescription doses. Nevertheless, for a large retroperitoneal IMT in close proximity to critical organs such as the bowel and kidneys, delivering a curative radiation dose while avoiding severe radiation-induced toxicity presents a significant clinical challenge. Herein, we report a case of such a challenging scenario, in which a modified dose-escalation VMAT technique was successfully employed. Through precise spatial dose avoidance, this approach achieved complete tumor regression while preserving the function of adjacent critical organs. This case aims to present a novel strategy for individualized, precise radiotherapy in locally advanced IMT.

## 2 Case description

A 59-year-old female presented with a 10-day history of fever accompanied by lumbar and abdominal pain. Physical examination revealed deep tenderness in the right lower quadrant, without rebound tenderness or muscular guarding.

### 2.1 Diagnostic assessment

**Figure 1:**
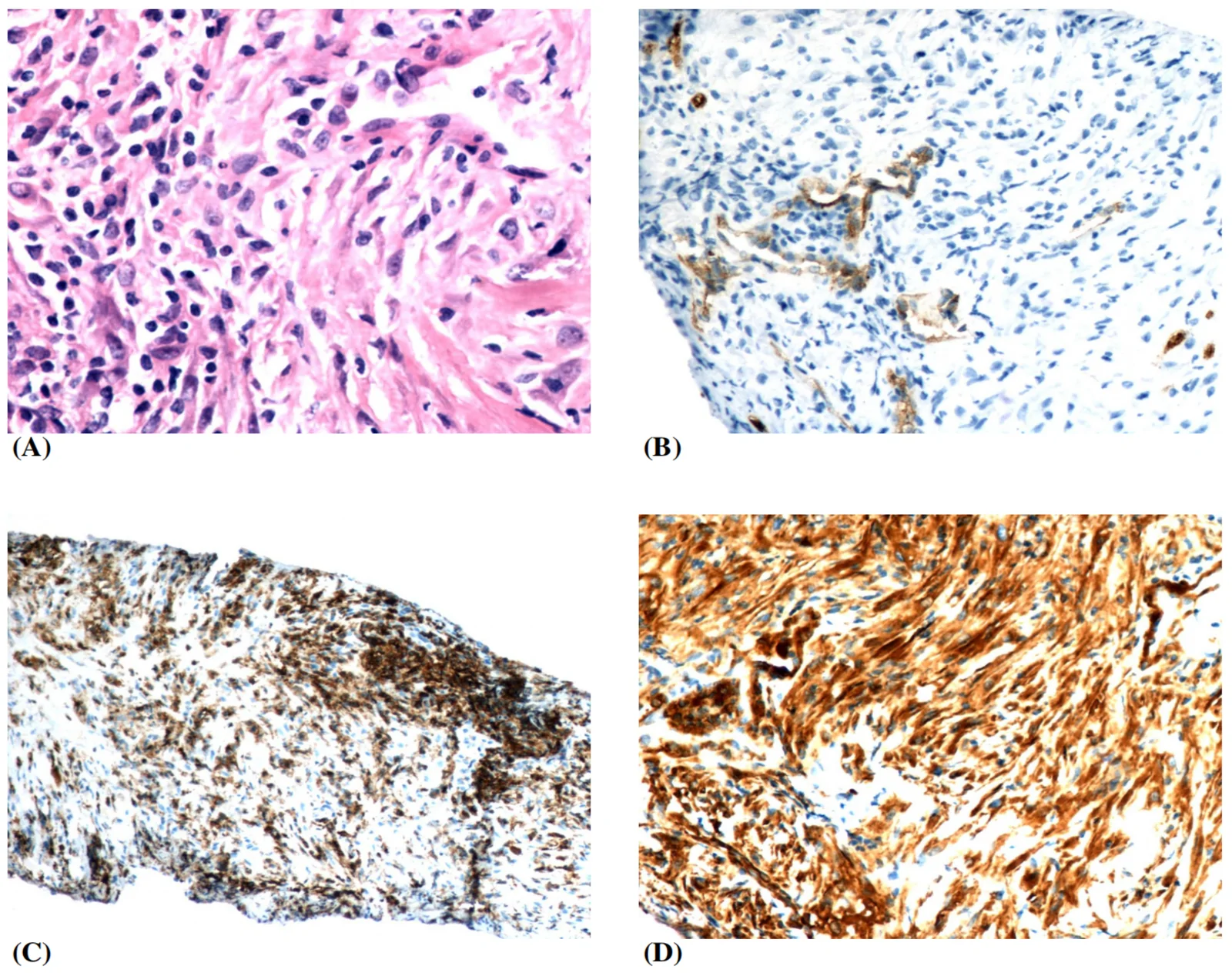
**(A)** Haematoxylin and eosin staining (HE), medium magnification (×40): Spindle cells proliferate in a fascicular pattern, with scattered inflammatory cells interspersed among the lesional cells. **(B)** Immunohistochemistry, medium magnification (×20): The lesional cells are negative for CD34, while vascular endothelial cells show positive staining. **(C)** Immunohistochemistry, medium magnification (×20): Scattered inflammatory cells show positive staining for leukocyte common antigen (LCA/CD45), while the lesional spindle cells are negative. **(D)** Immunohistochemistry, medium magnification (×20): The lesional cells exhibit diffuse strong cytoplasmic positivity for *α*-smooth muscle actin (*α*-SMA).

Laboratory tests revealed a white blood cell count of 21.06 × 10^9^/L. Contrast-enhanced computed tomography (CT) of the abdomen demonstrated a retroperitoneal mass measuring 9.2 × 6.3 × 6.0 cm, with heterogeneous enhancement and evidence of invasion into the right ureter and inferior vena cava. An ultrasound-guided percutaneous needle biopsy of the retroperitoneal mass was performed. Pathological examination confirmed a diagnosis of IMT (**Figure 1**). Histologically, the lesion was composed of fascicular spindle cells with scattered inflammatory infiltrates. Immunohistochemically, leukocyte common antigen (LCA/CD45) staining was restricted to scattered inflammatory cells (lesional cells negative); the lesional cells were positive for *α*-smooth muscle actin (*α*-SMA), and negative for CD34. These features were consistent with IMT. Capture-based next-generation sequencing (NGS) was performed using a panel covering key actionable drivers—including ALK, NTRK1/2/3, BRAF, KRAS, EGFR, ERBB2, MET, and RET—for the detection of point mutations, fusions, and copy number alterations. No class I actionable alterations were identified; two low-frequency PTEN class II variants were detected, with no approved IMT-targeted agents.

### 2.2 Intervention and treatment

**Figure 2:**
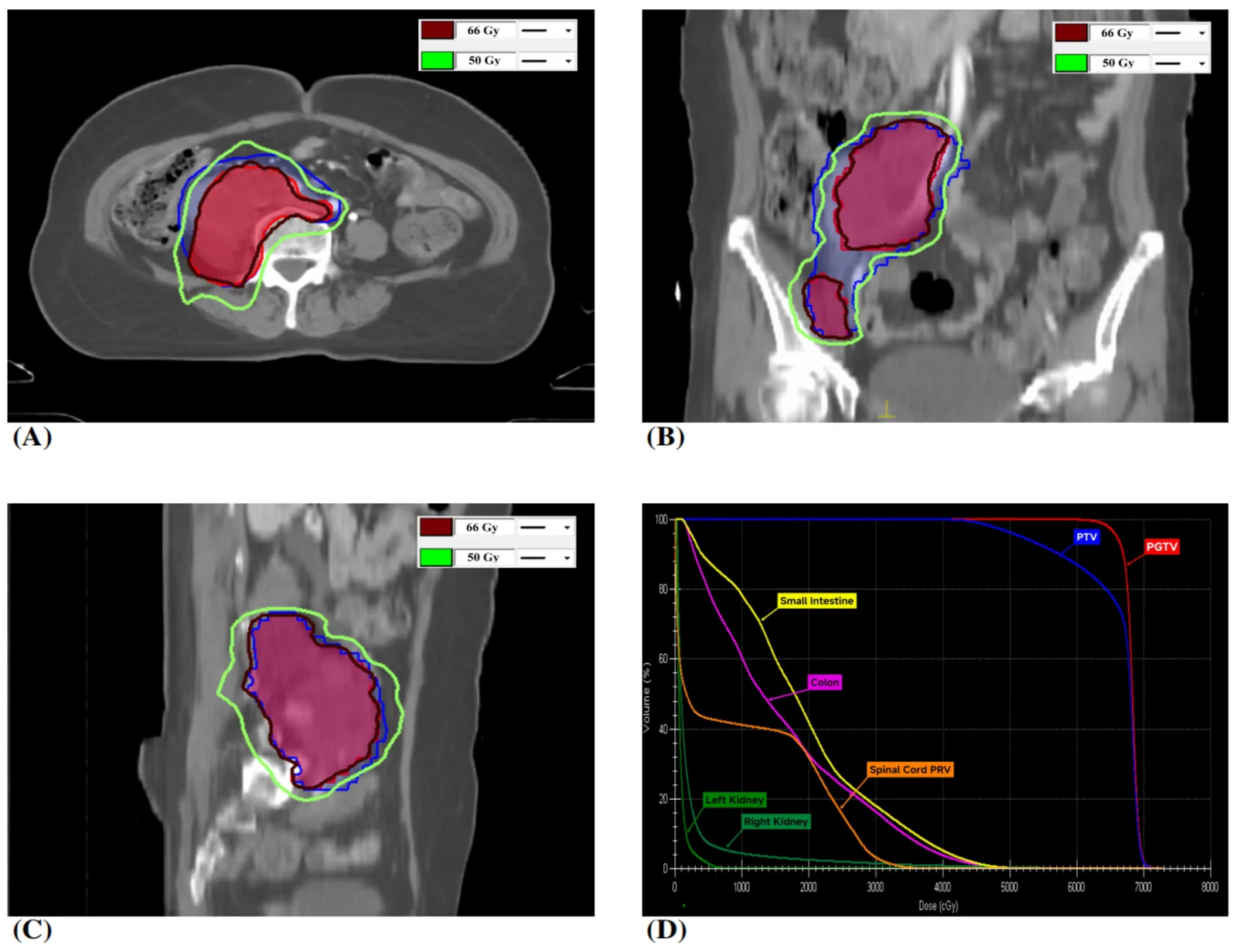
Radiotherapy plan for retroperitoneal IMT. **(A)** Axial CT slice showing PGTV (red) and PTV (blue). **(B)** Coronal CT slice showing PGTV (red) and PTV (blue). **(C)** Sagittal CT slice showing PGTV (red) and PTV (blue). **(D)** DVH showing dose distribution of target volumes and organs at risk.

Given the considerable tumor size and its intimate involvement with adjacent iliac vessels, the right ureter, and the iliopsoas muscle, curative-intent surgical resection was deemed highly challenging. Following anti-inflammatory therapy with corticosteroids, the patient’s symptoms, including fever and low back pain, showed suboptimal improvement. Given the lack of feasible surgical and targeted treatment options, the patient was treated with definitive volumetric modulated arc therapy (VMAT) using an Elekta Versa HD linear accelerator equipped with an Agility multileaf collimator. Daily online cone-beam computed tomography (CBCT) with a full-fan scan protocol (120 kV) was performed before each fraction, and images were co-registered with the planning CT using gray-value matching to correct setup errors. The gross tumor volume (GTV) was delineated based on pretreatment contrast-enhanced CT images, and two planning target volumes were designed: the conventional planning target volume (PTV) was generated by expanding the GTV by 5 mm, while the boost planning target volume (PGTV) was created by selectively shrinking the PTV by 7 mm toward hollow organs such as the small intestine and rectum to spare these organs at risk (OARs) from the high-dose region. The PGTV was not generated directly from the GTV; rather, it was derived from the PTV by selective retraction toward hollow organs. Consequently, at interfaces adjacent to the small intestine and rectum, the 7-mm retraction exceeded the 5-mm PTV expansion, meaning the PGTV fell approximately 2 mm short of the GTV surface in those restricted sectors; however, this peripheral zone remained covered by the PTV and received the 50-Gy prescription dose for subclinical disease coverage. This approach enabled dose escalation to the tumor region while minimizing radiation exposure to adjacent organs at risk, thereby achieving a precise balance between high-dose target coverage and organ preservation. The prescribed radiation dose was 66 Gy in 33 fractions to at least 95% of the PGTV, with the additional requirement that at least 95% of the PTV received 50 Gy. **Figure 2** illustrates the target design and dose distribution of the radiotherapy plan. The final plan achieved a 95% dose coverage of the PGTV and a 96.24% coverage of the PTV receiving 50 Gy. OAR dose parameters are summarized in **Supplementary Table 1**.

### 2.3 Follow-up and outcomes

**Figure 3:**
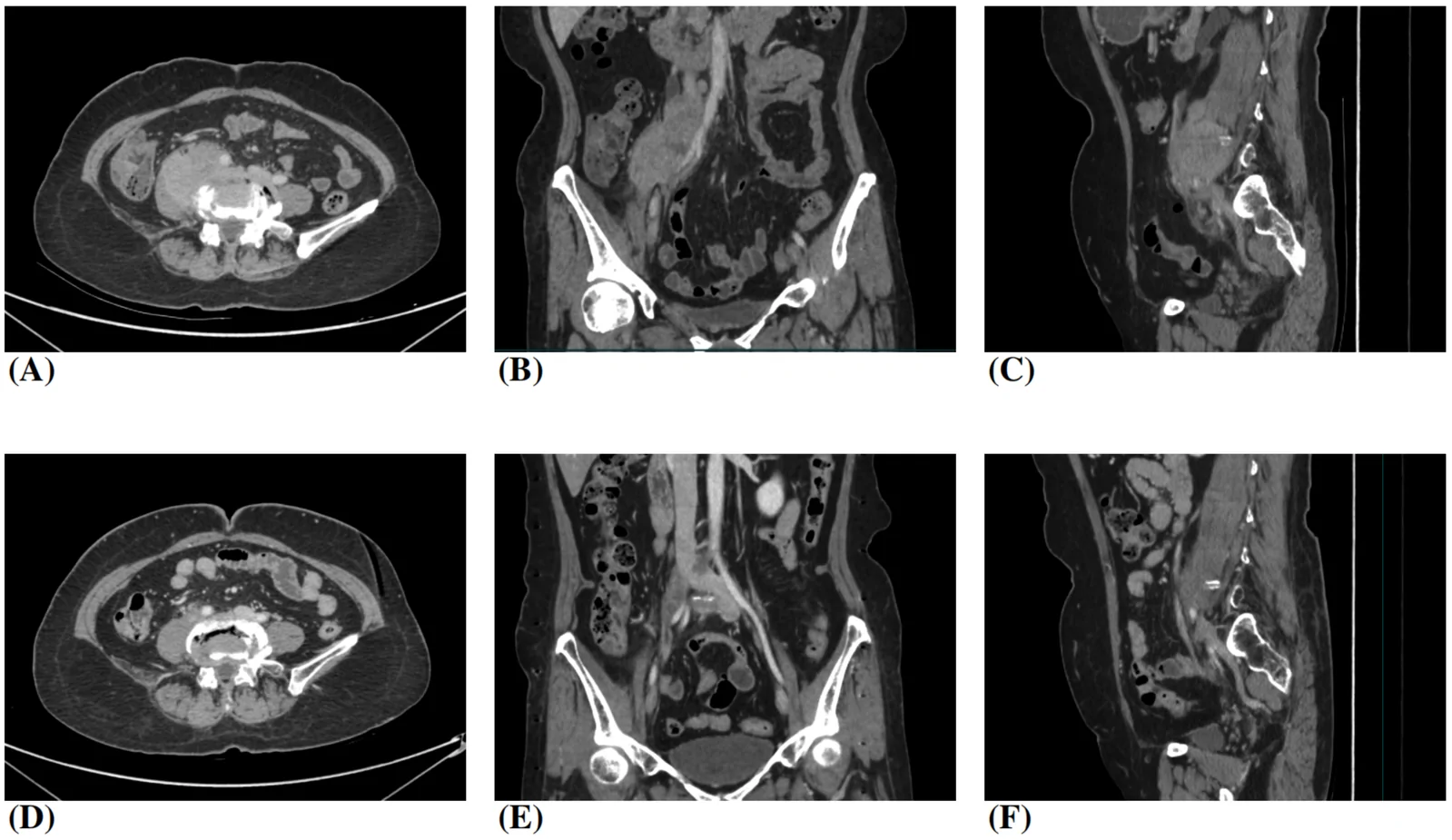
Pre- and post-radiotherapy CT comparison for retroperitoneal IMT. **(A)** Pre-radiotherapy axial CT slice showing the retroperitoneal IMT lesion. **(B)** Pre-radiotherapy coronal CT slice showing the retroperitoneal IMT lesion. **(C)** Pre-radiotherapy sagittal CT slice showing the retroperitoneal IMT lesion. **(D)** Post-radiotherapy axial CT slice showing the retroperitoneal IMT lesion. **(E)** Post-radiotherapy coronal CT slice showing the retroperitoneal IMT lesion. **(F)** Post-radiotherapy sagittal CT slice showing the retroperitoneal IMT lesion.

After approximately 10 fractions of radiotherapy, the patient’s symptoms, including fever and lumbar pain, showed significant relief, and had completely resolved by the end of treatment. No grade ≥2 radiotherapy- related adverse effects were observed per Radiation Therapy Oncology Group (RTOG) acute radiation toxicity criteria. Specifically, grade 1 lower gastrointestinal toxicity (mild lumbar and abdominal discomfort, not requiring analgesic intervention) and grade 1 leukopenia (lowest WBC 3.56 × 10^9^/L) were noted; grade 2 anemia (lowest hemoglobin 90 g/L) occurred; platelet count remained normal (lowest 184 × 10^9^/L) throughout. No upper gastrointestinal, dermatological, or genitourinary toxicity was observed. One month post-radiotherapy, contrast-enhanced CT revealed marked tumor shrinkage in the retroperitoneal region; the residual tissue was considered fibrotic, leading to an assessment of complete response (CR) (**Figure 3**). Subsequent follow-up was conducted every three months. At one year post-radiotherapy, asymptomatic right hydronephrosis was detected due to mid-ureteral stricture, and a ureteral stent was placed. At the time of stent placement, renal function was preserved (creatinine 86.60 µmol/L). At the 19-month follow-up, the stent remained in place and renal function was stable (creatinine 68.50 µmol/L). No evidence of tumor recurrence was observed. A timeline of key clinical events from symptom onset to the last follow-up is presented in **Table 1**.

**Table 1:** Timeline of clinical events.

Time point	Event
Day 1–10	Fever, lumbar and abdominal pain
Admission (initial work-up)	Physical exam revealing right lower quadrant tenderness; WBC 21.06 × 10^9^/L; CT showing 9.2 cm retroperitoneal mass invading right ureter and IVC
Biopsy	Ultrasound-guided percutaneous needle biopsy; pathological and immunohistochemical diagnosis of IMT
Genetic testing	Capture-based NGS: no class I actionable alterations
Initial treatment	Corticosteroid therapy: suboptimal symptomatic improvement
Radiotherapy (33 fractions, ∼6.5 weeks)	Dose-escalated VMAT (66 Gy/33 fr to PGTV, 50 Gy to PTV) with daily CBCT image guidance
During radiotherapy	Fever and lumbar pain significantly relieved after ∼10 fractions; complete symptom resolution by end of treatment
1 month post-RT	CT: marked tumor shrinkage, assessed as complete response (CR)
1 year post-RT	Asymptomatic right hydronephrosis due to mid-ureteral stricture; ureteral stent placed
19 months post-RT	Stent in place, renal function stable (creatinine 68.50 µmol/L), no recurrence

RT, radiotherapy; Gy, gray; fr, fraction; VMAT, volumetric modulated arc therapy; CT, computed tomography; WBC, white blood cell; IVC, inferior vena cava; IMT, inflammatory myofibroblastic tumor; NGS, next-generation sequencing; CBCT, cone-beam computed tomography; PGTV, boost planning target volume; PTV, planning target volume; CR, complete response.

## 3 Discussion

The abdominal cavity and retroperitoneal region represent the most frequently involved sites for extrapulmonary IMT, accounting for over 70% of cases (1). Affected patients may present with a variety of symptoms, including nausea, vomiting, melena, hematuria, abdominal or back pain, and gastrointestinal obstruction. These clinical manifestations typically result from tumor compression or infiltration of adjacent organs, leading to functional impairment. Due to its insidious onset, slow growth, and deep-seated location, IMT arising in the abdominal and retroperitoneal regions often remains clinically silent in the early stages. Symptoms usually only emerge when the tumor enlarges sufficiently to cause significant compression or invasion of nearby structures, prompting medical evaluation and subsequent detection. By this time, complete surgical resection may no longer be feasible, often limiting therapeutic options and leading to a poor prognosis.

For patients with IMT who are unable to tolerate or are not candidates for surgical resection, radiotherapy may serve as a definitive treatment modality (8). Given the low clinical incidence of IMT, there is currently no established consensus regarding the delineation of target volumes or the optimal prescribed dose. Existing literature consists primarily of case reports, with most employing radiation doses ranging from 45 to 60 Gy (**Supplementary Table 2**) (9–16). However, clinical experience with definitive radiotherapy for large IMT lesions remains scarce. Given the absence of IMT-specific dose-escalation data, we drew upon the established dose-response relationship in soft tissue sarcomas, for which definitive doses of at least 63–66 Gy are recommended. A systematic review of definitive radiotherapy for unresectable soft tissue sarcomas reported that doses of 64–66 Gy are associated with improved outcomes in large tumors (17); accordingly, we selected a prescribed dose of 66 Gy to the PGTV. While ensuring a high central dose to the tumor, careful consideration was given to the tolerance of adjacent normal tissues. A three-dimensional setup margin of 7 mm was applied to spare adjacent hollow organs such as the small intestine and colon, ensuring that the radiation dose remained within the safety constraints recommended by the RTOG (18). The conventional PTV received a dose of 50 Gy to provide adequate coverage of potential microscopic tumor infiltration, thereby reducing the risk of recurrence due to undertreated subclinical disease (19).

Higher-dose delivery necessitates more rigorous quality control (20). In the present case, daily CBCT was used for online image guidance to ensure accurate setup and delivery. Nevertheless, the current protocol has scope for further optimization. The current setup margin of 7 mm (three-dimensionally) from the hollow viscus was empirically determined based on clinical experience, which is inherently subjective and lacks a basis in individualized optimization derived from quantitative imaging or dose prediction. Future research could focus on the following improvements. First, pre-treatment four-dimensional computed tomography (4D-CT) can be employed to precisely characterize the motion trajectories of target volumes and OARs, providing a foundation for individualized internal target volume (ITV) delineation. Integration with respiratory gating techniques could further mitigate the impact of respiratory motion on abdominal organs, thereby reducing motion-induced uncertainties (21, 22). Second, image-guided adaptive radiotherapy (ART) strategies can be implemented to modify target volume boundaries and dose distributions in real time in response to tumor shrinkage or organ deformation during treatment, replacing fixed empirical retraction margins (23). The integration of these approaches would enable the transition from empirically determined spatial margins to a data-driven model based on individual patient anatomy and motion characteristics, thereby providing a more reliable quantitative foundation for clinical decision-making.

In this case, right-sided hydronephrosis and mid-ureteral stricture developed one year after radiotherapy. The stricture is most plausibly attributable to progressive fibrosis of the retroperitoneal tumor rather than primary radiation-induced ureteral injury, given the baseline ureteral invasion and the absence of acute ureteral toxicity during treatment. Nevertheless, a contributory role of radiation cannot be entirely excluded. Renal function remained stable and the ureteral stent remained in place at last follow-up. Surgical intervention would have been unable to achieve complete tumor resection, or would have necessitated removal of the entire right kidney and ureter. In contrast, radiotherapy achieved comprehensive dose coverage of the entire tumor, including subclinical lesions, thereby reducing the risk of recurrence. While attaining a curative outcome, radiotherapy also avoided the morbidities and risks associated with surgery, and more importantly, enabled organ preservation, ensuring the patient’s quality of life (24). Although ureteral stricture occurred, it was simply managed by ureteral stent placement.

The durability of our treatment strategy was substantiated by a progression-free survival of 19 months at the last follow-up. While this case achieved a successful outcome, its value as a single report in guiding radiotherapy for IMT remains inherently limited. Future efforts require the accumulation of additional cases, as well as the application of more advanced radiotherapy techniques such as proton therapy, to further enhance treatment efficacy and minimize normal tissue toxicity. Several take-home lessons emerge from this case. First, definitive radiotherapy with dose escalation to 66 Gy is feasible and can achieve durable complete response in large retroperitoneal IMTs when surgical and targeted options are unavailable. Second, spatial modulation of the boost volume—selectively retracting high-dose regions away from hollow organs—represents a practical strategy for balancing tumor coverage with OAR protection in the confined retroperitoneal space. Third, even when tumor control is achieved, ureteral stricture remains a potential late sequela for IMTs invading the ureter, underscoring the importance of long-term urological surveillance and proactive management of urinary complications. Finally, for IMTs lacking ALK or other actionable mutations, radiotherapy should be considered a definitive treatment option rather than merely a palliative measure. In conclusion, this case demonstrates that dose-escalated VMAT with spatial sparing of OARs is a feasible and effective definitive treatment for large, unresectable retroperitoneal IMT, achieving sustained complete response with acceptable toxicity. Further studies are warranted to define optimal radiation dose and fractionation regimens for IMT.

### 3.1 Patient perspective

I had a fever and bad pain in my back and belly, and I could barely do anything. I went to the hospital, and they found a tumor. They said surgery was not possible, so I thought, well, let’s just do radiotherapy—there wasn’t really any other good option anyway. During the radiotherapy, I just felt a bit tired, nothing else really bothered me. About a month later, the fever and pain went away, and a follow-up scan showed the tumor was gone. About a year later, they found a narrowing in my ureter, but a stent fixed it and it was fine. Now it’s been over a year and a half, I feel fine, my kidneys are good, and the tumor hasn’t come back. I’m quite satisfied that I was cured without surgery.

## Data Availability

The raw data supporting the conclusions of this article will be made available by the authors, without undue reservation.
