# Supplementary material for: Case Report: Dose-escalated volumetric modulated arc therapy achieving bowel and renal protection for large retroperitoneal inflammatory myofibroblastic tumor invading ureter and inferior vena cava
Source: Front Oncol. 2026 Aug 11;16:1818998. doi: 10.3389/fonc.2026.1818998 (PMC13504383; doi:10.3389/fonc.2026.1818998)
Supplement: Supplementary file 1 [file Table1.docx]

**Supplementary Tables**

References cited in this supplementary material are included in the main reference list.

**Supplementary Table 1. Organs at risk dose parameters**

| **Organ at Risk** | **Parameter** | **Value** |
| --- | --- | --- |
| Small intestine | D_max_  D_1cc_  V_45_ | 55.07 Gy  48.56 Gy  5.5 cc |
| Colon | D_max_  D_1cc_ | 56.44 Gy  48.87 Gy |
| Spinal cord PRV | D_max_ | 38.33 Gy |
| Right kidney | D_mean_  V_20_ | 2.43 Gy  2.38% |
| Left kidney | D_mean_  V_20_ | 0.91 Gy  0% |
| Right ureter^*^ | — | Not contoured |
| Inferior vena cava^*^ | — | Not contoured |

*Structures not delineated as separate OARs at the time of planning due to their intimate involvement with the GTV

**Supplementary Table 2. Case Reports of Radiotherapy for IMT.**

| **No.** | **Author** | **Tumor Location** | **Tumor Size** | **Radiotherapy Modality** | **Dose/f** | **Combined Therapy** | **Efficacy and Follow-up Outcome** |
| --- | --- | --- | --- | --- | --- | --- | --- |
| 1 | Maire et al. | Skull base | Not specified | Salvage radiotherapy | 20Gy/10f | Failed corticosteroid + RT | Clinical remission (3m); complete remission (2y) |
| 2 | Zhu et al. | Maxillary sinus, nasal cavity | Not specified | Salvage radiotherapy | Not reported | Corticosteroids + chemotherapy | Median follow-up 39m; 5-y survival 72% |
| 3 | Lisi et al. | Trachea | Not specified | Postoperative RT | 45Gy/25f | None | No recurrence (62m); no toxicity |
| 4 | Browne et al. | Left hilar region | 5.1×3.9 cm | Definitive radiotherapy | 45Gy/25f | None | Clinical remission (1m); minimal fibrosis (7.3y) |
| 5 | Biswas et al. | Right maxillary sinus | Not specified | Definitive radiotherapy | 60Gy/30f | Oral prednisolone | Complete clinical-radiological remission |
| 6 | Najjar et al. | Left upper lobe (lingular) | Not specified | Salvage radiotherapy | 4Gy/2f | None | Partial remission (3m); near-complete regression (6m) |
| 7 | Guo et al. | Right upper lobe + mediastinum | 7.2×6.7×6.1 cm | Postoperative RT | 54Gy/27f | Concurrent cisplatin chemo | No recurrence; PFS > 60m |
| 8 | He et al. | Multiple sites (nasal, arm, bladder) | 2.0–9.5 cm (max) | Postoperative RT | Not reported | Surgery ± chemotherapy | Most no recurrence; nasal IMT recurred/death |

RT, radiotherapy; Gy, gray; f, fraction; PFS, progression-free survival; m, month; y, yea
